# It's how you get there: walking down a virtual alley activates premotor and parietal areas

**DOI:** 10.3389/fnhum.2014.00093

**Published:** 2014-02-25

**Authors:** Johanna Wagner, Teodoro Solis-Escalante, Reinhold Scherer, Christa Neuper, Gernot Müller-Putz

**Affiliations:** ^1^Laboratory of Brain-Computer Interfaces, Institute for Knowledge Discovery, BioTechMed, Graz University of TechnologyGraz, Austria; ^2^Department of Biomechanical Engineering, Delft University of TechnologyDelft, Netherlands; ^3^Rehabilitation Clinic Judendorf-StrassengelJudendorf-Strassengel, Austria; ^4^Department of Psychology, BioTechMed, University of GrazGraz, Austria

**Keywords:** neurorehabilitation, robotic gait training, locomotion, motor planning, electroencephalography, interactive feedback, gait adaptation

## Abstract

Voluntary drive is crucial for motor learning, therefore we are interested in the role that motor planning plays in gait movements. In this study we examined the impact of an interactive Virtual Environment (VE) feedback task on the EEG patterns during robot assisted walking. We compared walking in the VE modality to two control conditions: walking with a visual attention paradigm, in which visual stimuli were unrelated to the motor task; and walking with mirror feedback, in which participants observed their own movements. Eleven healthy participants were considered. Application of independent component analysis to the EEG revealed three independent component clusters in premotor and parietal areas showing increased activity during walking with the adaptive VE training paradigm compared to the control conditions. During the interactive VE walking task spectral power in frequency ranges 8–12, 15–20, and 23–40 Hz was significantly (*p* ≤ 0.05) decreased. This power decrease is interpreted as a correlate of an active cortical area. Furthermore activity in the premotor cortex revealed gait cycle related modulations significantly different (*p* ≤ 0.05) from baseline in the frequency range 23–40 Hz during walking. These modulations were significantly (*p* ≤ 0.05) reduced depending on gait cycle phases in the interactive VE walking task compared to the control conditions. We demonstrate that premotor and parietal areas show increased activity during walking with the adaptive VE training paradigm, when compared to walking with mirror- and movement unrelated feedback. Previous research has related a premotor-parietal network to motor planning and motor intention. We argue that movement related interactive feedback enhances motor planning and motor intention. We hypothesize that this might improve gait recovery during rehabilitation.

## 1. Introduction

Gait recovery is a major rehabilitation goal in post-stroke therapy. Impairments in normal gait affect balance, stride length, walking speed, obstacle avoidance and endurance. These factors often lead to an increased risk of falls and related injuries (Said et al., 1999). In consequence, affected individuals are not able to react adequately and promptly to demands within their environment, which hinders them in performing activities of daily living autonomously (Duncan et al., 1998).

Much has been discussed about optimal training strategies in rehabilitation and different therapy approaches. Several key features including the form and intensity of motor training are assumed to support neural plasticity in motor learning. In gait rehabilitation extensive training can be provided by using a robotic gait orthosis that allows a high number of movement repetitions (Lum et al., 2002; Mehrholz et al., 2013). However, robotic rehabilitation alone generates a highly repetitive and monotonous practice environment that requires little effort from the individual. Findings on discrete upper limb movements indicate that active performance in the training is more effective for motor learning (Lotze et al., 2003; Kaelin-Lang et al., 2005). Furthermore several studies suggest that the individual's motivation in the training is one of the critical factors in determining the therapy outcome (Maclean and Pound, 2000; Liebermann et al., 2006). It has been argued that a more interactive and demanding learning context, might enhance the individual's motivation and promote active participation in the motor task. Virtual Environments (VEs) provide a convenient solution to these ends as different kinds of motor tasks with various degrees of difficulty can easily be implemented (Holden, 2005; Liebermann et al., 2006). Recent studies suggests that VE can in fact promote active participation during robotic gait training. Brütsch et al. (2010, 2011) and Schuler et al. (2011) showed that training with VE significantly increased active participation during robot assisted gait in children with various neurological gait disorders and healthy controls. Active participation was assessed using biofeedback values from hip and knee torques (Brütsch et al., 2010, 2011) and electromyographic activity of the lower limbs (Schuler et al., 2011). Other research suggests that VE combined with robot assisted lower limb training has a greater effect on improving gait parameters such as balance, speed, and endurance in individuals after stroke than robot-assisted training alone (Jaffe et al., 2004; You et al., 2005; Mirelman et al., 2009, 2010).

However, so far the underlying neurophysiological processes that are elicited by motor related feedback in a VE during gait training and their relevance to the relearning of motor skills have not been investigated. Active participation and voluntary drive in movements have been shown to be crucial for motor learning (Lotze et al., 2003; Kaelin-Lang et al., 2005). But how does the notion of voluntary drive translate to the movement of gait? In general voluntary movements have been defined as two different kinds of subjective experiences: “intention” which relates to the phase of movement planning and “agency” describing the feeling that one's own movement has caused a specific effect (Tsakiris et al., 2010). These feelings can be promoted by feedback in a VE. Findings also indicate that the experience of agency is related to the presence of perceptual and sensory feedback about the effects of motor actions in the physical world (Blakemore et al., 2002). Thus the feeling of agency can be increased by enhancing feedback to motor actions in a VE. Investigations on upper limb movements reveal a sensorimotor network of premotor-parietal cortices that is related to motor awareness and intention (Sirigu et al., 2003; Berti et al., 2005; Tsakiris et al., 2010), (for a review see Haggard, 2008). However, walking is a rhythmic and highly automated movement and it is not clear which parts of the movement are controlled by the cortex, the brain stem and central pattern generators in the spinal cord (Armstrong, 1988; Grillner et al., 1998). Hence motor awareness and intention most likely differ between walking and discrete upper limb movements. In animals motor areas of the cortex are only activated during gait initiation and gait adaptation, but not during unperturbed gait (Armstrong, 1988; Drew et al., 2008).

Few studies in humans have investigated motor preparation during gait. Recently we compared active to passive walking in a gait robot and found a trend for differences in sensorimotor EEG rhythms over the premotor cortex additionally to differences over sensory areas (Wagner et al., 2012). Wieser et al. (2010) studied evoked potentials related to gait like movements during an upright position. They found that the cortical activity over sensorimotor areas was highest shortly before a change of direction between the flexor and extensor movement of the legs. Haefeli et al. (2011) showed an increased activation over prefrontal areas during the preparation and performance of obstacle steps with EEG. Recently Sipp et al. (2013) showed that walking on a balance beam elicited increased electroencephalographic theta band activity over a wide range of mostly midline cortical areas compared to steady state treadmill walking. Several fNIRS studies have investigated motor preparation during gait. Increased activity over the prefrontal cortex (PFC) and the SMA was observed during adaptive walking compared to steady state walking (Suzuki et al., 2004), as well as during the preparation before gait initiation (Suzuki et al., 2008; Koenraadt et al., 2013). Additionally Koenraadt et al. (2013) found increased activation over the PFC during precision stepping. Consequently it seems that adaptive and challenging training paradigms that continually require participants to adjust their gait are necessary to produce motor planning during gait.

In the current study we examined the impact of an interactive VE feedback task on the EEG patterns during robot assisted walking. We compared this to walking with a visual attention task in which the stimuli were unrelated to the movement and mirror feedback where participants were observing their own movements. We chose these control conditions for two different reasons. First, to account for the amount of visual attention that is required by the interactive feedback task. The visual attention task provides visual stimuli unrelated to the movement, while the mirror feedback consists of visual information relevant to the participants' movement. The latter condition should thus activate the mirror neuron system and account for possible activations of this system during VE feedback. Higher cortical activation during VE compared to mirror feedback and the visual attention task should therefore reflect additional motor planning and visuomotor processing required by the interactive feedback. The second reason we chose the mirror feedback as a control conditions is that in automated gait rehabilitation therapy mirror feedback is often used. Research has demonstrated that mirror feedback during therapy can improve motor recovery after stroke (for a review see Ramachandran and Altschuler, 2009). These studies assume that part of the efficacy of mirror feedback could be due to the stimulation of dormant “mirror neurons.” Thus we wanted to examine whether the interactive VE feedback would produce a measurable higher activation of sensorimotor areas relative to mirror feedback.

In particular we hypothesize that walking with interactive feedback in a VE would increase motor planning and intention and thus activate premotor and parietal areas relative to walking with mirror feedback and a visual attention task. Additionally we hypothesize that if the VE task would yield higher cortical activation of these areas compared to mirror feedback interactive VE feedback may be more beneficial for motor learning.

## 2. Materials and methods

### 2.1. Participants

Eleven healthy volunteers (26 ± 2 years, 7 male) with no past or current neurological or locomotor deficits participated in this study. The experimental procedures were approved by the ethical committee of the Medical University Graz. Written informed consent was obtained from all subjects before the experiment.

### 2.2. Experimental design and procedure

**Figure 1:**
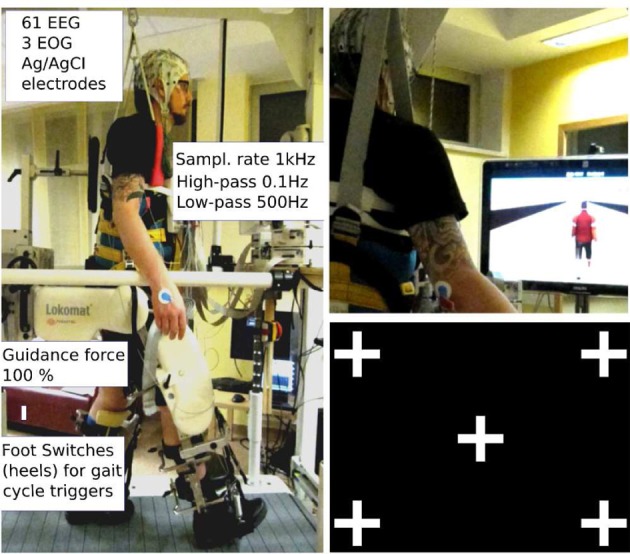
**Experimental setup: subject walking in the lokomat gait orthosis with body weight support**. The amplifiers for EEG recordings are fixed on a board in front of the participant. The orthosis is adapted and fixed to the participant's legs with the help of an experienced physical therapist; Left: robotic assisted walking. Speed (≤2.2 km/h) and body weight support (~30%) were adjusted for each participant; Right top: participant walking in the 3rd person VE condition. Right bottom: gaze screen with possible locations for the graphical objects.

Participants walked with a robotic gait orthosis (Lokomat, Hocoma AG, Switzerland) under five different visual feedback conditions. Each condition lasted 4 min and was repeated two times during the experiment. The Lokomat is a robotic driven gait orthosis that includes electrical drives in knee and hip joints and incorporates a motorized treadmill and body weight support system. Parameters of the Lokomat were adjusted according to the common practice in clinical therapy with the help of experienced physical therapists. Walking speed was adjusted according to the participants leg length with the formula: speed = 0.54 (leg) / 27.8 where leg is the participant's leg length in cm and the speed is computed in kilometer per hour. Walking speed ranged from 1.8 to 2.2 km per hour between participants. For comparison, fast overground walking speed lies at around 5 km/h (Bohannon, 1997). Body weight support (BWS) was adjusted for each participant at around 30%. The Lokomat was run in a control mode with 100% guidance force. The feedback conditions consisted of:

**NoFB** Participants walked while looking at a black screen.
**GAZE** Participants looked at white graphical objects sequentially appearing (for 3 s) in different locations on a black screen (see Figure 1).
**MIRROR** Participants watched themselves in a mirror while walking in the orthosis.
**3rdP VE and 1stP VE** Participants walked in a 3D Virtual Environment in 3rd and 1st person view. The task consisted in steering an avatar down an alley without crashing into the walls marking the edge of the path. The movement of the avatar was controlled using the participant's kinematic information measured within the gait orthosis. Steering of the avatar depends on the force executed by the participant on the gait orthosis and is measured by force sensors within the Lokomat. We used the augmented performance feedback that is implemented as standard in the Lokomat (Hocoma AG, Switzerland).

One gait cycle was defined as the interval between two right leg heel contacts (one gait cycle lasted from 1.6 to 2.4 s depending on the participant's leg length). Before starting the experimental sessions subjects were asked to train under the virtual reality feedback conditions for some minutes to get used to the orthosis and to steering in the VE. After a short training period (about 3 min for each VE task), all subjects reported that they were able to control sufficiently well the VR. Conditions were randomized. In all conditions, participants were asked to look straight ahead, not to close their eyes for prolonged periods of time, and to blink normally. Figure 1 summarizes the experimental setup.

### 2.3. Data acquisition

The EEG was recorded from 61 sites using two 32-channel amplifiers (BrainAmp MR plus amplifiers, Brainproducts, Munich, Germany). Electrodes were mounted in an electrode cap (EasyCap, Germany) according to the 5% 10/20 system (Oostenveld and Praamstra, 2001). The electrooculagram (EOG) was recorded from three electrodes, two placed on the outer canthi of the eyes and one between the eyes on the forehead. Both EEG and EOG were referenced to the left mastoid, and ground was placed on the right mastoid. All electrode impedances were reduced below 10 kΩ before the recording. Three-dimensional electrode coordinates were measured on a screening day prior to the actual measurement with the Zebris Elpos system (Noraxon, USA). EEG and EOG was acquired with 1 kHz sampling rate, and band pass filtered between 0.1 and 500 Hz. The timing of the heelstrike of both legs was assessed using mechanical foot switches placed over the calcaneus bone at the foot sole of both feet.

### 2.4. EEG analysis

EEG data analysis was performed using Matlab 2012b (The MathWorks Inc., Natick, MA) and EEGLAB 11.0b functions (Delorme and Makeig, 2004).

In Wagner et al. (2012) we showed that it is possible to account for artifact contamination of the EEG with Infomax Independent Component Analysis during robotic gait training following the methods of Onton et al. (2006) and Gwin et al. (2010). Before submitting the EEG to an ICA the data was preprocessed accordingly.

First the data (EOG and EEG) were high pass filtered at 1 Hz using a zerophase FIR filter (order 7500) to minimize drifts, low pass filtered at 200 Hz (zerophase FIR filter order 36), and subsequently downsampled to 500 Hz. Channels with prominent artefacts were excluded from further analysis (avg. 2.2; range: 0–7), and the EEG and EOG were rereferenced to a common average reference that was computed from the remaining EEG channels. The continuous EEG data were then visually inspected for non-stereotyped artifacts (e.g., swallowing, electrode cable movements, etc.) and affected partitions were removed from further analysis. For automatic artifact rejection the data were partitioned into segments of 0.5 s to identify outliers exceeding the average of the probability distribution of values across the data segments by ±5 *SD*. On average, per condition 72% of the gait cycles of each participant's EEG data remained in the analysis (range: 61–89%, *SD*: 11).

Next, the preprocessed datasets containing EEG and EOG were decomposed using an adaptive independent component analysis (ICA) mixture model algorithm (AMICA) (Palmer et al., 2006, 2008). AMICA is a generalization of the Infomax algorithm (Bell and Sejnowski, 1995; Makeig et al., 1996) and multiple mixture (Lee et al., 1999; Lewicki and Sejnowski, 2000) ICA approaches. Infomax ICA utilizes temporal independence to perform blind source separation (Makeig et al., 1996). ICA was performed on individual subjects over all conditions (GAZE, MIRROR, 1stP VE, 3rdP VE, noFB).

Individual component scalp maps were submitted to a single dipole source localization algorithm using a standardized three-shell boundary element head model (BEM) implemented in EEGLAB (Oostenveld and Oostendorp, 2002; Delorme et al., 2012). Individual participants' electrode positions were co-registered and aligned with a standard brain model (Montreal Neurological Institute, MNI, Quebec, Canada). Ideally independent components representing synchronous activity within a cortical domain are characterized by scalp maps fitting the projection of a single equivalent current dipole. Therefore, the goodness of fit for modeling each independent component scalp map with a single equivalent current dipole was used to quantify component quality. Only ICs whose dipoles were located within the head and fitted their scalp projection with a residual variance of less than 10% were considered further.

ICs representing artifacts were identified and rejected from further analysis by visual inspection considering the scalp map, the event-locked time course and the power spectrum. The remaining ICs were submitted to an automatic clustering routine implemented in EEGLAB (Delorme and Makeig, 2004) using principal component analysis (PCA). Feature vectors coding differences between ICs in dipole location, power spectral density (PSD) (3–40 Hz), and scalp projection were reduced to 10 principal components and clustered with *k*-means (with *k* = 13). Components further than three standard deviations from the obtained cluster centers were moved to a separate “Outlier” cluster. Only clusters that contained more than half of the participants were further analyzed. Furthermore, as we were interested in motor related functions, we considered only clusters in sensorimotor areas.

### 2.5. Clusters of cortical ICs

**Figure 2:**
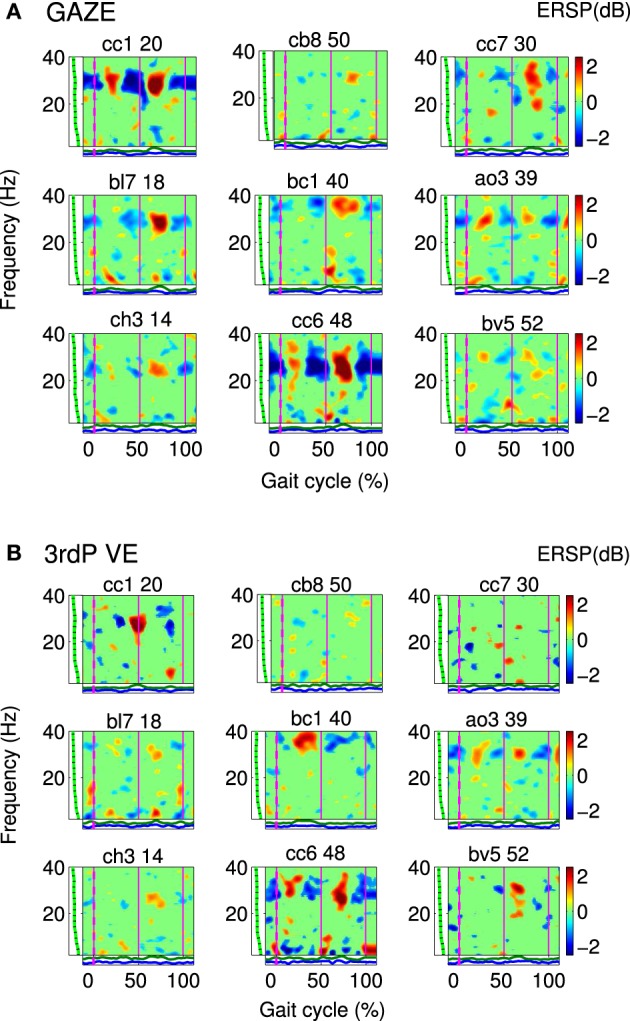
**Gait event-related spectral perturbation maps (ERSPs) for cluster A: Single IC plots showing significant changes in spectral power during the gait cycle for (A) GAZE and (B) 3rdP VE**. Non-significant differences relative to the full gait cycle baseline (*p* ≤ 0.05) are masked in green (0 dB). Vertical lines mark the temporally aligned events of right leg heel contact as the beginning (0%) and end (100%) of the gait cycle, and the left heel-strike (50%). The gait-cycle related modulation in the 23–40 Hz band is more pronounced during GAZE compared to 3rdP VE. The band in which this modulation appears varies over subjects and encompasses frequencies from 23 to 40 Hz. [The codes on top of the figures (e.g., cc1 20) represent participant codes (e.g., cc1), and the number of the IC (e.g., 20)].

The PSD (using Welch's Method) and event-related spectral perturbations (ERSP) (Makeig, 1993) were computed for each independent source. To generate gait cycle ERSPs single trial spectograms were computed and timewarped using a linear interpolation function, thus aligning the timepoints for right and left heelstrike over trials. Relative changes in spectral power were obtained by averaging the difference between each single-trial log spectogram and baseline (the mean IC log spectrum over all gait cycles per condition). To visualize significant event-related changes from baseline, deviations from the average gait cycle log spectrum were computed with a bootstrap method (Delorme and Makeig, 2004). This analysis revealed gait cycle related activity in one of the clusters that was significant from baseline (see Figure 2). This modulation occurred in a varying frequency band ranging from 23 to 40 Hz between persons. For further statistical analysis an individual band in this frequency range was selected for each participant, considering only frequencies that were significantly different from baseline. Spectral activity in 8–12 Hz alpha and 15–20 Hz beta bands did not differ overtly between subjects. Furthermore the spectra of single subjects did not show multiple peaks in these frequency bands. Therefore the standard bands were used for further analysis.

For statistical analysis ERSPs were computed for the GAZE, MIRROR, 1stP VE and 3rdP VE using a common baseline: the average gait cycle log spectrum computed from the noFB condition. Independent component ERSPs were then averaged in three frequency bands: 8–12 Hz (alpha), 15–20 Hz (beta), and subject specific bands in the range 23–40 Hz.

**Figure 3:**
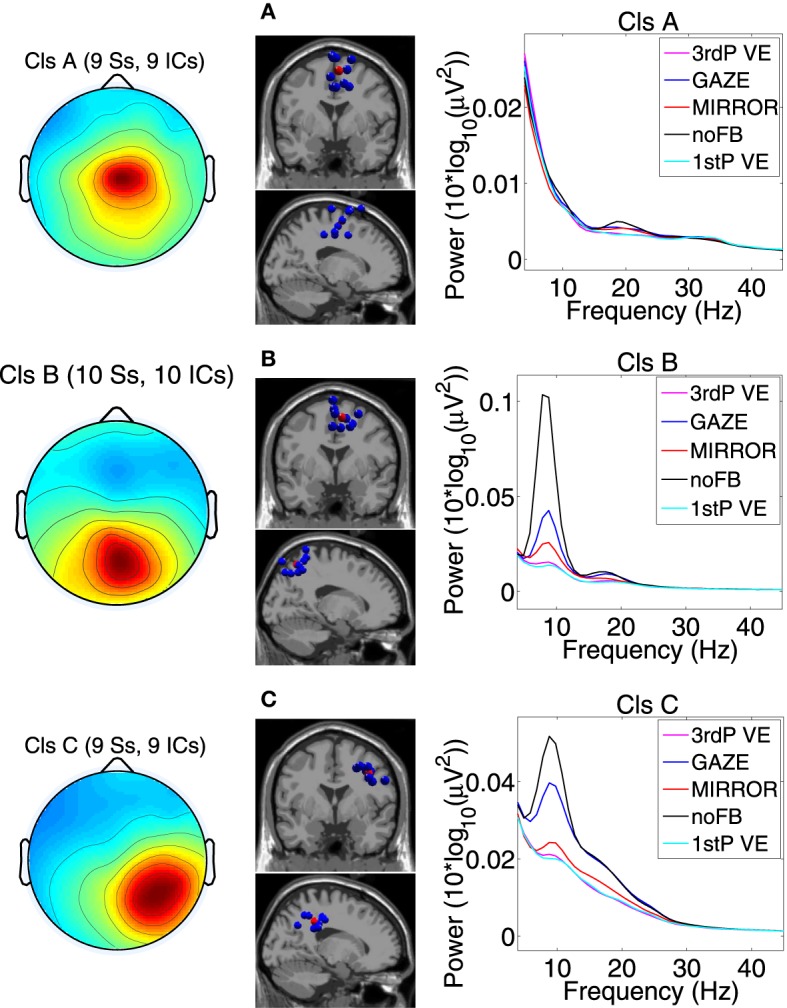
**Scalp projection, spatial location and power spectra of independent component clusters (A) Cluster A located in the supplementary motor area (premotor cortex); (B) Cluster B located in the posterior cortex (Brodmann area 7); (C) Cluster C located in the posterior cortex (Brodmann area 40)**. From left to right in each row: cluster average scalp projections; dipole locations of cluster ICs (blue spheres) and cluster centroids (red spheres) visualized in the MNI brain volume in coronal and sagittal views; PSD for all feedback conditions. For cluster B and C a clear difference in PSD between noFB and Gaze vs. both of the VE conditions in the mu and in the beta range can be observed [Naming: Ss, ICs—number of subjects (Ss) and Independent Components (ICs) in the cluster].

For statistical analysis we divided the gait cycle symmetrically in two stationary phases 10–30% and 60–80% of the gait cycle and two transition phases 30–60% and 80–10% of the gait cycle. Since two of the sensorimotor clusters we identified were located in midline areas we could not attribute their activity to one of the hemispheres (see Figure 3). The stationary phases correspond to the midstance (10–30%), initial swing (60–73%), and miswing phases (73–87%). The transition phases correspond to the terminal stance (30–50%), preswing (50–60%), terminal swing (87–100%), and loading response (0–10%) following the definition by Perry (1992).

A repeated measurements 4 × 4 within-subject ANOVA with factors “feedback” (GAZE vs. MIRROR vs. 1stP VE vs. 3rdP VE) and “gait cycle phase” (two stationary phases and two transition phases) was computed for each cluster and each frequency band separately. Multiple comparisons were corrected controlling for false discovery rate (Benjamini and Yekutieli, 2001) with a significance level set *a priori* at 0.05. In cases where the assumption of sphericity was violated significance values were Greenhouse-Geisser corrected. Additionally we computed the effect size η^2^. Simple paired *t*-tests with a bootstrapping method were employed for *post hoc* testing, and multiple comparisons were corrected controlling for false discovery rate with an *a priori* alpha level at 0.05. For *post hoc* comparisons we also computed the effect size (cohen's *d*) based on the distance between means.

## 3. Results

Three clusters located in central midline areas revealed differences between the feedback conditions (see Figure 3). The number of subjects and sources contained in each cluster and Tailarach coordinates of cluster centroids are displayed in Table 1.

**Table 1:** **Clusters of independent sources obtained with ICA**.

**Cluster**	**Location of cluster centroid (Brodmann area)**	**Tailarach coordinates (x,y,z)**	**Number of subjects (S) and ICs**
A	Supplementary motor area (BA6)	5, −1, 58	9 S, 9 ICs
B	Parietal cortex (BA7)	8, −56, 55	10 S, 10 ICs
C	Parietal cortex (BA40)	37, −35, 37	9 S, 9 ICs

**Figure 4:**
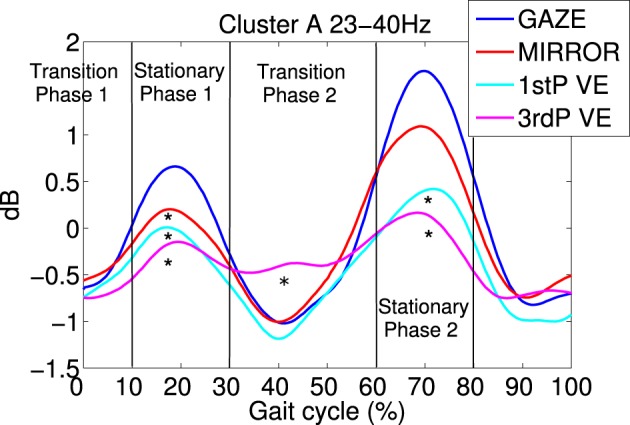
**Average gait event-related spectral perturbations (ERSPs) for cluster A:** for each feedback condition ERSPs are computed relative to the full gait cycle baseline obtained from the noFB condition. Then ERSPs are averaged over subject specific frequency bands between 23 and 40 HZ and then averaged over subjects for cluster A. Temporally aligned events are marked for the right leg heel contact at 0% as the beginning and 100% as the end of the gait cycle, and for the left heel-strike at 50%. Each feedback condition is represented by a colored trace. It is visible that during 1stP and 3rdP VE in stationary gait phases (10–30% and 60–80%) power in this band is decreased compared to the other feedback conditions. Also a difference between 3rdP VE and 1stP VE during the second transition phase of the gait cycle (30–60%) is evident. Vertical lines mark the beginning and the end of gait cycle phases. Asterisks mark significance between feedback conditions in the indicated gait cycle phase.

Cluster A, located in the premotor cortex, showed significant changes (*p* ≤ 0.05) from baseline relative to the phases of the gait cycle in the band 23–40 Hz visible in the single IC ERSPs during GAZE, NoFB, MIRROR and in reduced form during 1stP VE and 3rdP VE, (see Figure 2). This cluster also presented a significant difference in the average spectrum between the feedback conditions in the beta band (*F*_(3, 24)_ = 6.9 *p* ≤ 0.0094, η^2^ = 0.46), (see Table 2). *Post hoc* tests revealed a significant (*p* ≤ 0.03) difference between VE and all other feedback conditions. For gait cycle related modulations in the 23–40 Hz frequency range a significant interaction between gait phases and conditions was found (*F*_(9, 72)_ = 2.6, *p* ≤ 0.0094, η^2^ = 0.25)(see Table 3). *Post hoc* tests revealed that power in this range was significantly (*p* ≤ 0.0085) reduced in the two stationary gait phases during both of the VE conditions compared to GAZE (see Figure 4). But only the second stationary gait phase during 3rdP VE was significantly (*p* ≤ 0.0085) different from MIRROR. Compared to GAZE, MIRROR showed significantly (*p* ≤ 0.0085) reduced power in this band in the first stationary gait phase. Interestingly there is a significant difference between 1stP VE and 3rdP VE in the second transition phase of the gait cycle. For an overview and Cohen's *d* values see Table 4.

**Table 2:** **ANOVA results: significant main and interaction effects**.

	**Cluster A**	**Cluster B**	**Cluster C**
8–12 Hz		Feedback	Feedback
		*F*_(3, 27)_ = 9.9	*F*_(3, 24)_ = 10.0
		*p* ≤ 0.0094, η^2^ = 0.56	*p* ≤ 0.0094, η^2^ = 0.55
15–20 Hz	Feedback	Feedback	Feedback
	*F*_(3, 24)_ = 6.9	*F*_(3, 27)_ = 11.7	*F*_(3, 24)_ = 14.0
	*p* ≤ 0.0094, η^2^ = 0.46	*p* ≤ 0.0094, η^2^ = 0.60	*p* ≤ 0.0094, η^2^ = 0.64
23–40 Hz	Feedback x		Feedback
	Gait Phase		
	*F*_(9, 72)_ = 2.6		*F*_(3, 24)_ = 8.3
	*p* ≤ 0.0094, η^2^ = 0.25		*p* ≤ 0.0094, η^2^ = 0.51

**Table 3:** **Significant differences in mean gait cycle spectra between feedback conditions (*p* ≤ 0.05 corrected with false discovery rate), and effectsize (cohen's *d*) (*d*1 and *d*3, respectively denote Cohen's *d* values for 1stP VE and 3rdP VE)**.

	**Cluster A**	**Cluster B**	**Cluster C**
8–12 Hz		VE-GAZE	VE-GAZE
		(*d*1 = 1.30, *d*3 = 1.44)	(*d*1 = 1.48, *d*3 = 1.19)
		VE-MIRROR	MIRROR-GAZE
		(*d*1 = 1.05, *d*3 = 0.98)	(*d* = 1.11)
15–20 Hz	VE-GAZE	VE-GAZE	VE-GAZE
	(*d*1 = 1.09, *d*3 = 1.00)	(*d*1 = 1.57, *d*3 = 2.51)	(*d*1 = 2.11, *d*3 = 1.57)
	VE-MIRROR	VE-MIRROR	VE-MIRROR
	(*d*1 = 0.76, *d*3 = 0.74)	(*d*1 = 0.91, *d*3 = 0.81)	(*d*1 = 0.83, *d*3 = 0.69)
			MIRROR-GAZE
			(*d* = 1.21)
23–40 Hz	see Table 4		VE-GAZE
			(*d*1 = 1.65, d3 = 1.59)
			VE-MIRROR
			(*d*1 = 0.81, d3 = 0.64)

**Table 4:** **Significant differences in single gait phase spectra between feedback conditions (*p* ≤ 0.0085) and cohen's *d* values for Cluster A**.

	**MIRROR**	**1stP VE**	**3rdP VE**
GAZE	1st stationary gait phase	Stationary gait phases	Stationary gait phases
	*d* = 0.88	*d* = 0.63, *d* = 0.95	*d* = 0.89, *d* = 1.03
MIRROR			2nd stationary gait phase
			*d* = 0.65
3rdP VE		2nd transition gait phase	
		*d* = 0.56	

For cluster B (parietal cortex, Brodman area 7) the ANOVA revealed a significant main effect for the mean spectrum between the visual feedback conditions in the mu band (*F*_(3, 27)_ = 9.9, *p* ≤ 0.0094, η^2^ = 0.56), and in the beta band (*F*_(3, 27)_ = 11.8, *p* ≤ 0.0094, η^2^ = 0.60). *Post hoc* tests show that spectral power in the mu band (*p* ≤ 0.0025) and in the beta band (*p* ≤ 0.0045) is significantly reduced in the VE conditions compared to MIRROR and GAZE. The ANOVA for cluster C (parietal cortex, Brodmann area 40) revealed a significant main effect for the mean spectrum between the visual feedback conditions for the mu band(*F*_(3, 24)_ = 10.0, *p* ≤ 0.0094, η^2^ = 0.55), the beta band (*F*_(3, 24)_ = 14.0, *p* ≤ 0.0094, η^2^ = 0.64) and the gamma band (*F*_(3, 24)_ = 8.3, *p* ≤ 0.0094, η^2^ = 0.51) (see Figure 3). *Post hoc* tests show that spectral power in the mu band (*p* ≤ 0.0055) is significantly reduced in the VE conditions and in the MIRROR condition compared to GAZE. The *post hoc* tests also show that spectral power in the beta band (*p* ≤ 0.013) and in the 23–40 Hz range (*p* ≤ 0.0075) is significantly reduced in the VE conditions compared to MIRROR and GAZE. Additionally the tests reveal that during MIRROR feedback spectral power in the beta band (*p* ≤ 0.013) is significantly reduced compared to GAZE. For an overview of significant comparisons and Cohen's values refer to Tables 2 and 3.

## 4. Discussion

Our analysis revealed three independent component clusters in premotor and parietal areas that showed significantly decreased spectral power in alpha, beta and 23–40 Hz frequency ranges during the interactive VE tasks compared to MIRROR and GAZE. This spectral power decrease indicates a higher neuronal activation (Pfurtscheller and Lopes da Silva, 1999).

Gait cycle related modulations in cluster A visible in the single IC ERSPs (see Figure 2) showed reduced activity during 3rdP VE compared to GAZE. Statistical analysis revealed that during both VE conditions power in the 23–40 Hz range is significantly decreased in the two stationary gait phases compared to GAZE. Also comparisons between MIRROR vs. GAZE and MIRROR vs. VE show only significant differences in stationary gait phases. Interestingly, however, there is a significant difference between 1stP VE and 3rdP VE in the second transition phase of the gait cycle (see Figure 4 and Table 4). In a previous study we found the same gait cycle related modulation in a 25–40 Hz frequency range during active and passive robot-assisted walking in the premotor cortex (Wagner et al., 2012). Central midline activity in the frequency range 30–45 Hz has been previously related to muscle activation during upper and lower limb movements (Pfurtscheller and Neuper, 1992; Pfurtscheller et al., 1993; Brown, 2000; Mima et al., 2000; Alegre et al., 2003; Müller-Putz et al., 2003, 2007; Raethjen et al., 2008). Results from Pfurtscheller and Lopes da Silva (1999) and Pfurtscheller et al. (1996) suggest that activity in an overlapping frequency band is involved also in motor planning. These studies reported synchrony of oscillations in the frequency range 36–40 Hz over the premotor area and in relation to the sensorimotor area shortly before movement-onset and during execution of movement. Interestingly Petersen et al. (2012) recently observed synchrony in the frequency range 24–40 Hz between EEG recordings over the foot motor area and the electromyogram from the tibialis anterior muscle during steady state walking. The significant coupling occurred prior to heel strike during the swing phase of walking. This corticomuscular coherence is similar in frequency band and cortical location to the gait cycle related modulation we find in the 23–40 Hz range. The stationary gait phases in our study coincide with the swing phases of both legs. Hence the decreased power during VE may represent processes involved in motor planning during these phases. The difference between 1stP VE and 3rdP VE during the second transition phase of the gait cycle is especially interesting and may indicate that participants were using different strategies for steering the avatar in the two conditions. We generally observed a more variable pattern of the 23–40 Hz modulation during 3rdP VE compared to the other conditions.

Our results also show a significant decrease in beta band power in the premotor cortex during VE compared to MIRROR and GAZE. Numerous scalp EEG and ECoG studies have related event-related desynchronization (ERD) in the alpha (8–13 Hz) and beta (15–25 Hz) rhythms to the activation of sensorimotor areas (Crone et al., 1998; Pfurtscheller and Lopes da Silva, 1999; Neuper and Pfurtscheller, 2001; Pfurtscheller et al., 2003; Miller et al., 2007), while synchrony in alpha and beta bands has been connected to a deactivation or inhibition of these areas (Klimesch et al., 2007; Neuper et al., 2007). Interestingly two recent studies showed that elevated synchrony in the sensorimotor beta rhythm promotes postural and tonic contraction and causes movements to be slowed (Gilbertson et al., 2005; Joundi et al., 2012); and a recent review suggests that modulation of beta activity is predictive of potential actions (Jenkinson and Brown, 2011). There is evidence that these principles hold for whole body movements such as walking. Wieser et al. (2010) showed decreased alpha and beta band power during gait like leg movements in an upright position, compared to periods of rest in which participants were lying. Presacco et al. (2011) showed that spectral power in the alpha band is suppressed during precision walking compared to standing. These results are in line with our recent study where we showed that alpha and beta spectral power in sensorimotor areas is suppressed during robot assisted walking compared to standing (Wagner et al., 2012). We also show that spectral power in these bands is significantly decreased during active compared to passive walking. Thus our findings indicate that the task of active gait adjustment in the VE requires enhanced motor planning and increases activity in the premotor cortex. This is in line with numerous studies that relate increased activity in the premotor area to the planning of single limb movements (Pfurtscheller and Berghold, 1989; Ikeda et al., 1992; Tanji, 1994), (for a review see Haggard, 2008). Recent studies have demonstrated that the premotor areas are also activated during gait initiation and adaptation (Suzuki et al., 2004, 2008; Haefeli et al., 2011; Koenraadt et al., 2013).

In the posterior parietal cortex (PPC) two clusters were identified. One located centrally (Cluster B) and one located in the right hemisphere (Cluster C). In Cluster B power in the mu and beta band was significantly suppressed during both VE conditions compared to MIRROR and GAZE. Cluster C also revealed decreased power in the beta band and the 23–40 Hz range during the VE tasks relative to all other feedback conditions. The 23–40 Hz range is overlapping with the upper beta band, and is suppressed during feedback conditions in which participants had to actively modify their steps. We assume therefore that a decrease in this band has the same functional meaning previously described for the mu and beta band. Alpha and beta rhythms in the parietal cortex have been previously linked to spatial attention, decision making, and sensorimotor integration (Capotosto et al., 2009; Donner and Siegel, 2011; Hipp et al., 2011; Capotosto et al., 2012). Interestingly two recent studies by Tombini et al. (2009) and Perfetti et al. (2011) relate alpha and beta ERD in parietal regions to the movement planning in visually guided upper limb movements under both feedforward and feedback control. For the MIRROR condition a significant power decrease in mu and beta bands relative to the movement unrelated feedback (GAZE) was observed solely in Cluster C. The PPC has been related to the mirror neuron system (Fogassi et al., 2005), we therefore conclude that the activation we find during MIRROR feedback is related to the participants' monitoring of their own movements.

Our results show that parietal cortex regions are more activated in conditions that require visually guided gait adaptation. These results are in line with studies that associate the PPC with visuomotor transformations in reaching movements. Neuronal recordings in monkeys have identified two subareas in the PPC responsible for the action planning of different body parts: the lateral intraparietal area (LIP) for saccades and the parietal reaching region (PRR) for reaching (Snyder et al., 1997). In humans, functional magnetic resonance imaging (fMRI) studies on the PPC have determined regions corresponding to the monkey PRR area (Connolly et al., 2003; Pellijeff et al., 2006). Recently Wang and Makeig (2009) demonstrated that it is possible to decode intended movement direction using human EEG recorded over the parietal cortex with a delayed saccade-or-reach task. Neuronal recordings in cats have revealed a higher activation in the PPC during visually guided gait modification, and suggest that the PPC may contribute to locomotor control (Drew et al., 2008). Interestingly a recent study has related activity in the parietal cortex directly to the awareness of human actions (Desmurget et al., 2009). Previous findings also indicate that the PPC is involved in the planning of eye-movements (Snyder et al., 1997). Planning of eye-movements in our study should have occurred mainly during GAZE as subjects were supposed to direct their gaze to objects appearing in different corners of the screen. In the parietal clusters we can observe decreased power in mu and beta bands during GAZE compared to NoFB (see Figure 3). Possibly some of this activity is related to the planning of eye-movements. However, differences between GAZE and VE should reflect the portion of activity not related to saccades.

Our findings that an interactive gait adaptation task activates premotor and parietal areas is especially interesting as these areas have been related to motor intention and motor planning (Haggard, 2008). The increased activity we find in premotor and parietal areas during walking in a VE might thus reflect increased motor planning that is required by the adaptive training paradigm. VE feedback elicited a higher activation compared to movement unrelated feedback and mirror feedback in all of the clusters. Mirror feedback showed enhanced activation relative to movement unrelated feedback only in one of the parietal clusters. This provides evidence that the benefits of gait training with a more demanding and interactive task may be superior to simple mirror feedback.

Interestingly we found a significant difference between 1stP VE and 3rdP VE in the premotor cortex during one of the transition phases of the gait cycle. In general 3rdP VE seems to be related to a more variable pattern of the 23–40 Hz modulation compared to the other conditions, including 1stP VE. This could be an indication that the gait movements are less regular and less automatic involving more motor planing during 3rdP VE compared to 1stP VE, at least during certain phases of the gait cycle. Studies on body ownership show that first person perspective is superior to third person perspective VE for the induction of full-body ownership illusions (Slater et al., 2010; Petkova et al., 2011). These studies relate the first person and third person perspective, respectively to an egocentric and allocentric reference frame. Studies show that the processing of egocentric spatial information and self-motion activates the right parietal cortex (Maguire et al., 1998; Andersen et al., 1999; Vogeley and Fink, 2003). Interestingly in our study we found clusters only in the right parietal cortex, and these were more activated during the VE walking tasks compared to MIRROR and GAZE. However, we did not find differences between 1stP and 3rdP VE in these clusters. Differences between 1st and 3rdP perspective were located in the premotor cortex, a brain region that has been identified in a previous study to be related to the feeling of agency (Tsakiris et al., 2010). From observations we can say that the participants in our experiment needed more time in the beginning to get used to the first person control in the VE. We could speculate that this increased performance success in visuomotor adaptation might have induced a greater feeling of agency in the third person perspective.

Our results further support previous findings (Brütsch et al., 2010, 2011; Schuler et al., 2011) suggesting that a more challenging gait adaptation task can promote the motivation for active participation in the movement. It is, however, not clear to which extent this motivation is increased by the immersiveness of the VE or whether any kind of interactive feedback might have the same effect. A recent study by Zimmerli et al. (2013) suggests that the interactivity of the training environment is fundamental in promoting the participants' active engagement in the motor task. Interactivity can be enhanced by providing functionally significant responses to the movement.

## 5. Conclusion

This study is the first to analyze brain activity during an interactive visual gait adaptation task with a robotic gait orthosis, and to show that the premotor and parietal areas are involved in visually guided gait in humans. We found that mu, beta, and lower gamma rhythms in premotor and parietal cortices are suppressed during conditions that require an adaptation of steps in response to visual input. Such suppression indicates increased activation of these brain areas. We show that this activity is higher compared to mirror feedback and a visual attention task. Higher cortical activation during visually guided gait adaptation may reflect additional motor planning and visuomotor processing. Activity in the parietal cortex likely reflects direct visuomotor transformations required by the task. Increased activity in the premotor cortex may indicate motor planning involved in adapting the steps to the visual input. Considering studies showing that voluntary drive is crucial for motor learning (Lotze et al., 2003; Kaelin-Lang et al., 2005), our results suggest the possible benefit of goal directed walking tasks that recruit brain areas involved in motor planning. Our results are relevant for gait rehabilitation after stroke and may help to better understand the cortical involvement in human gait control.

### Conflict of interest statement

The authors declare that the research was conducted in the absence of any commercial or financial relationships that could be construed as a potential conflict of interest.
